# Mental representation of the locations and identities of multiple hidden agents or objects by a bonobo

**DOI:** 10.1098/rspb.2025.0640

**Published:** 2025-08-20

**Authors:** Luz Carvajal, Christopher Krupenye

**Affiliations:** ^1^ Department of Psychological & Brain Sciences, Johns Hopkins University, Baltimore, MD, USA

**Keywords:** mental representation, social cognition, agent representation, tracking location, object tracking, primate, bonobo, social intelligence hypothesis

## Abstract

Humans are adept at navigating the social world in part because we flexibly map the locations and identities of agents around us. While field studies suggest primates can track individual conspecifics, controlled experiments are needed to determine the complexity of this capacity and isolate the underlying representations. Across five object-choice tasks, we show that our closest relative, a bonobo (Kanzi), can concurrently track the locations and identities of multiple (specifically, two) hidden agents (Experiment 1), that this capacity deploys mental representations rather than tracking agents’ last observed locations (Experiment 2), and that these representations can integrate visual or auditory signatures of identity (Experiment 3). Finally, we show that this bonobo performs similarly on an analogous multiple-object tracking invisible displacement task (Experiments 4–5), consistent with multiple agent- and object-tracking potentially recruiting common representational machinery. This work uncovers the rich representations of the social world that are shared by humans and other apes.

## 1. Introduction

The social world presents unique cognitive challenges for social animals. It comprises numerous familiar (and sometimes unfamiliar) individuals, each acting based on their own goals, knowledge and social ties. According to the social intelligence hypothesis, the challenges of navigating such a dynamic environment create selective pressures that favour cognitive mechanisms that render the social world more predictable [1–4]. While researchers have amassed decades of observational data on the social behaviour of group-living animals, we still know much less about the knowledge those animals possess about their social worlds [5] and, in particular, the representational building blocks that undergird this knowledge. Human social knowledge is built on the ability to generate enduring cross-modal representations of familiar individuals that capture visual and vocal signatures of an individual’s identity [6]. We also mentally represent the whereabouts of familiar individuals and groups that are not in our immediate perceptual environment, allowing us to maintain group cohesion, reason about others’ future behaviour and make decisions about how best to interact with (or avoid) them [7]. Critically, we know surprisingly little about how widely these representational capacities are distributed across other animals and if they evolved millions of years ago in a common ancestor that humans share with other great apes.

Identifying the shared evolutionary foundation of human social knowledge requires research on our very closest relatives, bonobos (*Pan paniscus*) and chimpanzees (*Pan troglodytes*). These species live in large societies, containing dozens and sometimes even hundreds of familiar individuals, and range in dense forests where social partners often move out of view. Their fission–fusion dynamics also mean that their community regularly splits into smaller foraging parties with changing compositions [8]. Accordingly, apes would benefit from cognitive capacities for tracking social partners across space and time.

Bonobos and chimpanzees can recognize the faces and vocalizations of familiar conspecifics, even after years apart [9–14]. Chimpanzees have also been shown to recognize familiar humans, even those wearing masks [15]. There is reason to believe that some animals have modality-independent representations of other agents [16–18]. However, just one chimpanzee and no bonobos have so far been tested for the capacity to match the vocalizations and faces of familiar individuals [19–21].

Identifying other individuals is particularly useful for prediction and decision-making if representations of individual agents can be linked to other information, such as the agent’s whereabouts. Wild howler monkeys can recognize their neighbours’ identity and location [22] through vocalization, but they might achieve this from long-term exposure to stable locations of neighbour groups. Wild vervet monkeys can track the transient location and ongoing displacement of at least one conspecific, generating expectations about that agent’s location even when it is out of sight [23]. Nonetheless, male Guinea baboons did not show evidence of tracking a familiar female in a similar protocol [24], suggesting that not all primates are able or motivated to track the displacements of their groupmates. Critically, no such studies have tested apes’ ability to track agents’ whereabouts. Moreover, while field studies have yielded seminal insights into this capacity [22–24], studies in more controlled contexts are necessary to precisely specify the underlying representations and determine, for example, whether animals can track more than one agent at a time.

Captivity may also shape agent tracking in ways that present opportunities for research. For example, captive individuals are in routine contact with human caregivers, who are of high social relevance but who also can be instructed to behave in more standardized ways than conspecifics, allowing for highly controlled experimental investigation of agent-tracking and of whether animals extend their agent-tracking capacities beyond conspecifics. In captivity, it is also possible to fully control visual and auditory access to familiar agents. Finally, captivity allows us to directly compare, on analogous paradigms, animals’ ability to track agents as well as other entities, like objects, shedding light on whether these capacities may rely on a common cognitive mechanism for object representation or instead reflect independent mechanisms with distinct properties and limitations.

Interestingly, controlled captive studies have demonstrated that apes can reliably track the location of a hidden object, including through displacements [25–30], but that they struggle to track more than one object at a time [26,27]. This pattern of results, coupled with the fact that we do not yet know whether primates (or other animals) can track more than one agent at a time, leaves open multiple possibilities. First, it is possible that primates may have only limited abilities to track multiple entities, regardless of whether they are objects or agents. Alternatively, methodological demands may have hampered primates’ performance in past object-tracking tasks, and once these demands are alleviated, we may find that they are capable of multiple-object and perhaps also multiple-agent tracking. Finally, it may be that observed limitations are real but are specific to object tracking, especially if object-tracking and agent-tracking recruit different mechanisms. Indeed, there is some reason to think that human infants have different modes of construal for self-propelled goal-directed agents relative to objects [31]. Thus, it is possible that the apparent quantity limitation on invisible object tracking in apes might be unique to an object-tracking system, and can be overcome when the content of the representation is an agent. While this specific question has not yet been studied in primates, another line of work on infants suggests socially relevant content or context allows infants to perform above previously observed representational limitations, either by representing more types of objects or a higher quantity [32]. This performance benefit, deriving not only from agentic content but even from an agentic context, may suggest that agents are simply more salient and motivating than objects, rather than that they are tracked by a distinct system. On a related note, it has been shown that apes’ memory is better for events involving social models, an effect that is independent from attention [33]. In sum, socially relevant stimuli could be processed differently in domains like memory and object representation, either through distinct mechanisms or simply as more salient stimuli. From an evolutionary perspective, an improved capacity for agent tracking in bonobos would also make sense considering the high cognitive demands of their social organization, and the greater ecological cost of losing track of a group member relative to an object [23]. Taken together, comparisons across analogous agent-tracking and object-tracking tasks are critical to identify whether apes can represent multiple agents or objects and whether these capacities appear to recruit common or differentiable mechanisms.

In summary, it remains unknown what representations and mechanisms subserve agent and object tracking in non-human animals and whether humans are unique in their capacity to mentally represent the whereabouts and identities of multiple familiar agents (or objects). To address these gaps, we designed a novel series of controlled experiments to examine bonobos’ capacity to mentally represent the locations of familiar agents, and to test several hypotheses about the underlying mechanisms. Across tasks, two people each hid behind one of three human-sized opaque barriers. We challenged a bonobo to indicate their locations after hiding (Experiment 1), after an invisible displacement (Experiment 2) or after auditory (but no visual) information was provided (Experiment 3). Finally, we tested the bonobo’s ability to track inanimate objects under an analogous paradigm (Experiments 4 and 5).

## 2. General methods

### (a) Participants

Across all tasks, we tested Kanzi, a 42-year-old male bonobo housed at the Ape Cognition and Conservation Initiative in Des Moines, Iowa, USA. Kanzi’s social group included three other males and three females. These bonobos participate in cognitive testing regularly, and are never deprived of food or water. They are separated in small groups as part of their regular husbandry procedures, and Kanzi was always alone in a familiar section of the facility during testing. Participation in this study was voluntary: Kanzi was free to approach and leave the experimental set-up at will. Other bonobos in the same institution also began the training: we conducted three training sessions (of up to 12 trials each) with Nyota, two with Maisha and Mali, and 11 with Teco. With limited time and resources to conduct the study, we abandoned training with Nyota and Maisha because they never began pointing towards visible humans, and with Teco and Mali because, although they showed some human-directed pointing, they (unlike Kanzi) showed little improvement in pointing specifically at the human depicted in a photo prompt, before the end of our research trip. This study received ethical approval from the Animal Care and Use Committees at Johns Hopkins University (protocol no. PR22A408) and at Ape Initiative (protocol no. 232605-01).

Kanzi presented a unique and powerful opportunity to address our question in a much more straightforward way than would be possible with almost any other ape in the world. This is because, as one of the last surviving enculturated apes from the ape language studies, he exhibited not only strong engagement with cognitive tasks but also rich forms of communication with humans—including pointing, use of symbols, and response to spoken English [34–36]—that we could exploit in the design of our task.

### (b) Materials

**Figure 1.**
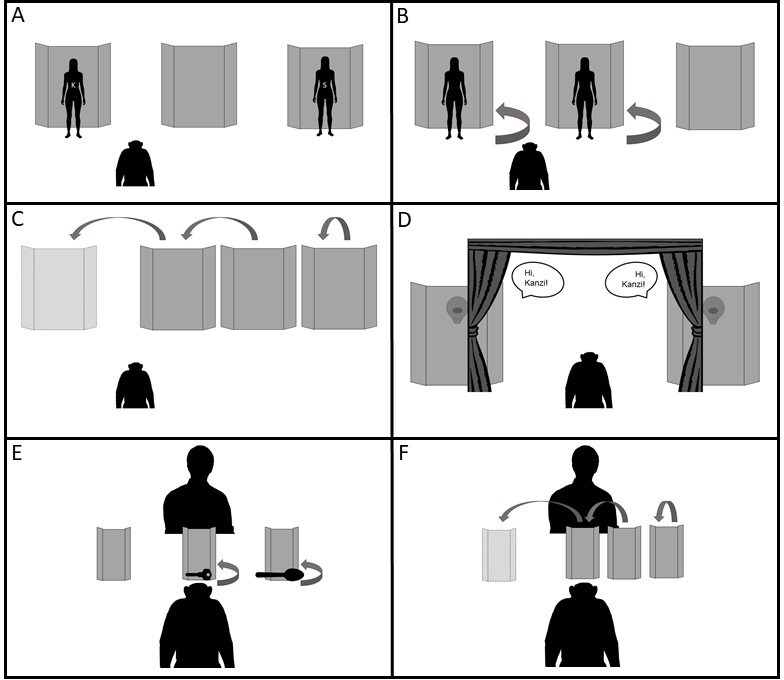
Participant’s view of the set-up across experiments. (A) Training: Kanzi was tasked with pointing at whichever of two visible caregivers matched a photo prompt. (B) Experiment 1: Kanzi observed two caregivers hide behind different barriers, and was then shown a photo prompt and tasked with pointing at whichever barrier contained the depicted caregiver. (C) Experiment 2: this procedure was identical to Experiment 1 except that all barriers were displaced following caregiver hiding and before the photo prompt. (D) Experiment 3: a curtain allowed two caregivers to surreptitiously hide behind different barriers. Caretakers then spoke in turn as the photo prompt was shown to Kanzi, and again Kanzi was tasked with pointing to the location containing the depicted caretaker. (E) Experiment 4: mirroring Experiment 1, Kanzi viewed two objects (a key and spoon) being hidden behind different barriers, and was then shown a photo prompt and tasked with pointing at whichever barrier contained the depicted object. (F) Experiment 5: this procedure was identical to Experiment 4 except that all barriers were displaced following object hiding and before the photo prompt. See also electronic supplementary materials, videos S1–S5.

In each task (figure 1A–F), a pair of familiar caregivers (Experiments 1−3: always the same two individuals) or objects (Experiments 4−5: spoon and key [34]) were hidden behind cardboard barriers and then the subject was shown a roughly life-sized colour photo (electronic supplementary material, appendix 1) of one of the hidden agents (20 × 25 cm photos of caregiver faces) or objects (8 × 10 cm photos). A spoon and key were chosen as the objects for Experiments 4−5 based on a past study showing that Kanzi was particularly familiar with these objects and could match spoken labels with images of them [34]. The barriers (1.88 m for Experiments 1−3 and 32 cm for Experiments 4−5) were winged at the side and had brown cloth at the bottom to ensure full occlusion of agents/objects from different angles. In Experiment 3, an opaque black curtain on a black metal frame 1.98 m high and 3.05 m wide was placed in front of the barriers to obscure which barrier each agent went behind. Caregivers then projected their voice through a cardboard cone 1.52 m high on the barriers. Cameras recorded all trials, and high-value foods (banana, apple, grape, peanuts) were used as rewards.

### (c) Coding and analysis

For 100% of trials, Kanzi’s choices were independently coded by Experimenter 1 (E1) (live, and then double checked from video), and by a second reliability coder (from video) who was blind to the research question and to the correct answer in each trial. Side of pointing was decided based on arm/hand position in relation to Kanzi’s body, and the coders used the visual aid provided by Kanzi’s enclosure, which consists of metal bars forming a grid that helped standardize hand/arm position (see electronic supplementary materials, video S1–S5). The reliability coder received one muted 2−3 s long clip for each experimental trial, consisting only of Kanzi’s point with no information of the location of Experimenters 2 and 3 (E2 and E3), and no footage of E1’s live response. Inter-rater agreement across experiments was high (Cohen’s kappa = 0.983, *z* = 23.4, *p* < 0.001). In the very few instances of disagreement, we defaulted to E1’s coding for analysis. All analyses were performed in R version 4.4.0 [37] and RStudio version 2024.04.1. For each experiment, we used a two-tailed binomial test to test whether Kanzi’s performance exceeded that expected by chance. We tested a variety of alternative explanations (table 1) using binomial tests as well as logistic regressions using the glm and glmer functions from the lme4 package [39]. All data and analysis scripts are publicly available at https://osf.io/q2a8d/.

**Table 1.** Alternative explanations and their predicted pattern of results in Experiments 1, 2 and 5. Full model results are in electronic supplementary materials, tables S2–S8.

alternative explanation	expected results	test used	findings exp 1	findings exp 2	findings exp 5
learning	improvement as trials progress	mixed effects logistic regression model with correct response as outcome, and trial as a fixed effect (full results in electronic supplementary materials, tables S2–S5)	no effect of trial, estimate = 0.003 (95% CI = −0.027 to 0.033), *p* = 0.838	no effect of trial, estimate = 0.071 (95% CI = −0.077 to 0.225), *p* = 0.35	no effect of trial, estimate = −0.024 (95% CI = −0.07 to 0.008), *p* = 0.117
tracking only one agent/object (consistently)	one of the two people/objects will be tracked accurately, but not the other	separate two-tailed binomial tests for each person/object (test proportion = 0.33)	tracking both: E2 17/30 correct, *p* = 0.011 (95% CI = 0.374–0.745) E3 18/30 correct, *p* = 0.003 (95% CI = 0.406–0.773)	trend towards tracking both: E2 15/30 correct, *p* = 0.079 (95% CI = 0.312–0.687) E3 18/30 correct, *p* = 0.003 (95% CI = 0.406–0.773)	trend towards tracking both: key 15/30 correct, *p* = 0.079 (95% CI = 0.313–0.687) spoon 16/30 correct, *p* = 0.031 (95% CI = 0.343–0.717)
tracking only one agent/object (inconsistently) AND choosing randomly between the two remaining barriers	majority of errors being on empty barrier (tracking of only one agent/object would yield correct performance when that is the prompted agent/object, and when the tracked one was not the prompted agent/object, Kanzi would choose randomly between the two remaining barriers, one of which in fact contains the correct agent/object, so it would only register as an error when he chooses the empty one)	one-tailed binomial test with incorrect trials only, testing probability of pointing at empty barrier (test proportion = 0.5)	majority of errors NOT on empty barrier (7/25 errors on empty barrier), *p* = 0.993 (95% CI = 0.139−1)	majority of errors NOT on empty barrier (9/27 errors on empty barrier), *p* = 0.974 (95% CI = 0.186−1)	majority of errors NOT on empty barrier (10/29 errors on empty barrier), *p* = 0.969 (95% CI = 0.2−1)
ignoring empty barrier and choosing randomly between the two barriers with agents/objects	performance at chance when he does not point at empty barrier	two-tailed binomial test with trials where he did not point at empty barrier only (test proportion = 0.5)	above chance even when ignoring empty (35/53 correct when not pointing at empty barrier), *p* = 0.027 (95% CI = 0.517–0.785)	above chance even when ignoring empty (33/51 correct when not pointing at empty barrier), *p* = 0.049 (95% CI = 0.501–0.776)	at chance when ignoring empty (31/50 correct when not pointing at empty barrier), *p* = 0.119 (95% CI = 0.472–0.753), though performance still high (62% correct)
ignoring empty barrier AND tracking only one agent/object	worse performance when empty barrier is in the middle position (harder to exclude from attention if central to visual field) (e.g. [38])	logistic regression with correct response as outcome and empty barrier in the middle as predictor (full results in electronic supplementary materials, tables S6–S8)	no effect of empty barrier in the middle, estimate = −0.82 (95% CI = −1.94 to 0.268), *p* = 0.142	no effect of empty barrier in the middle, estimate = 0.619 (95% CI = −0.471 to 1.769), *p* = 0.274	no effect of empty barrier in the middle, estimate = 0.201 (95% CI = −0.877 to 1.296), *p* = 0.715

### (d) General procedure

All tasks involved a consistent structure, with precise timing and choreography: Kanzi was alone in the testing room, separated from experimenters by a mesh barrier. E1 sat in the testing area and attracted Kanzi to the starting location. Once the agents (E2 and E3) or objects (spoon and key) were in place and Kanzi had been provided visual (Experiments 1, 2, 4, 5) or auditory (Experiment 3) information about their location, E1 showed Kanzi a photograph of one of the two agents or objects for 5 s while saying ‘This is [agent/object name]’. E1 then removed the photo and asked Kanzi ‘Where is [agent/object name]?’ Kanzi typically pointed to the photo prompt while it was visible, so the removal of the photo prompt and the verbal request to identify the location of the depicted individual marked the start of the choice phase. During the choice phase, E1 remained still, looking forward with a neutral expression, to avoid providing any cues. Kanzi’s first point during the choice phase was scored as his choice. Once Kanzi pointed at a location, E1 indicated that Kanzi had made a choice, the contents of both locations were revealed and, if correct, Kanzi was immediately rewarded (in Experiments 1−3, by the prompted caregiver directly). Accepted pointing behaviours were: pointing with arms or hands, with or without extended fingers, towards one of the barriers. If Kanzi produced an ambiguous point, E1 would ask ‘Which one?’ until Kanzi moved his hand/arm to a clearer position. If on any trial Kanzi did not respond in 30 s, agents and objects returned to their starting locations and the trial was repeated (which happened only five times over the course of all experiments). While Experiment 3 only featured two barriers, Experiments 1, 2, 4 and 5 included a third barrier that was empty and served as a distractor to prevent Kanzi from making accurate choices without tracking both agents simultaneously (e.g. by tracking one and making an inference-by-exclusion). Location and identity of the two agents or objects (and of the empty barrier) were counterbalanced and pseudorandomized within each session. Every experiment consisted of five sessions of 12 trials each for a total of 60 trials. Kanzi participated in no more than two sessions back-to-back and no more than five in a day (though typically just two sessions per day), with all consecutive sessions corresponding to the same experiment, and no more than one experiment being conducted in one day.

#### (i) Training stage

Before experimental trials began, Kanzi needed to understand that this task works like a multimodal match-to-sample: that upon viewing a photo prompt, he needed to point to the depicted real-life agent or object. To achieve this, we first trained him to do this with agents or objects in full view. In each trial, two agents or objects were initially located in front of the middle barrier and then moved to their corresponding locations in front of one of the three barriers, with the individual slated for the left-most location moving first. All other details of the procedure (e.g. regarding prompting, timing, point-scoring) followed the general procedure. Kanzi advanced to the test phase once he pointed to the correct person or object for 10 out of 12 trials per session over two consecutive sessions. Kanzi required 12 sessions to pass training with agents and, later, two sessions to do so with objects. Seven of these agent training sessions involved the pair of agents on which Kanzi achieved criterion, who were then used as the experimental agents in Experiments 1−3. We continued agent training for six additional sessions beyond Kanzi’s passing on the first pair, such that Kanzi had 11 agent training sessions involving pairs of agents that he did not master and that were therefore not used in Experiments 1−3. Although we imagine that Kanzi would have ultimately mastered these pairs with sufficient practice, we discontinued training and advanced to Experiments 1−3 after 18 training sessions, to ensure sufficient time remained in our research trip to complete the experiments. Details of Kanzi’s performance during training sessions and the agents on each of them are available on electronic supplementary material, figure S1.

#### (ii) Experiment 1: hidden agents

#### Methods

The procedure was identical to training, except once E2 and E3 were standing in front of their corresponding barrier, one at a time, they said ‘Hi, Kanzi’ and moved behind their barrier, starting with the left-most person always. Prompting and reactions to Kanzi’s responses followed the same guidelines as the general procedure.

#### Results and discussion

**Figure 2.**
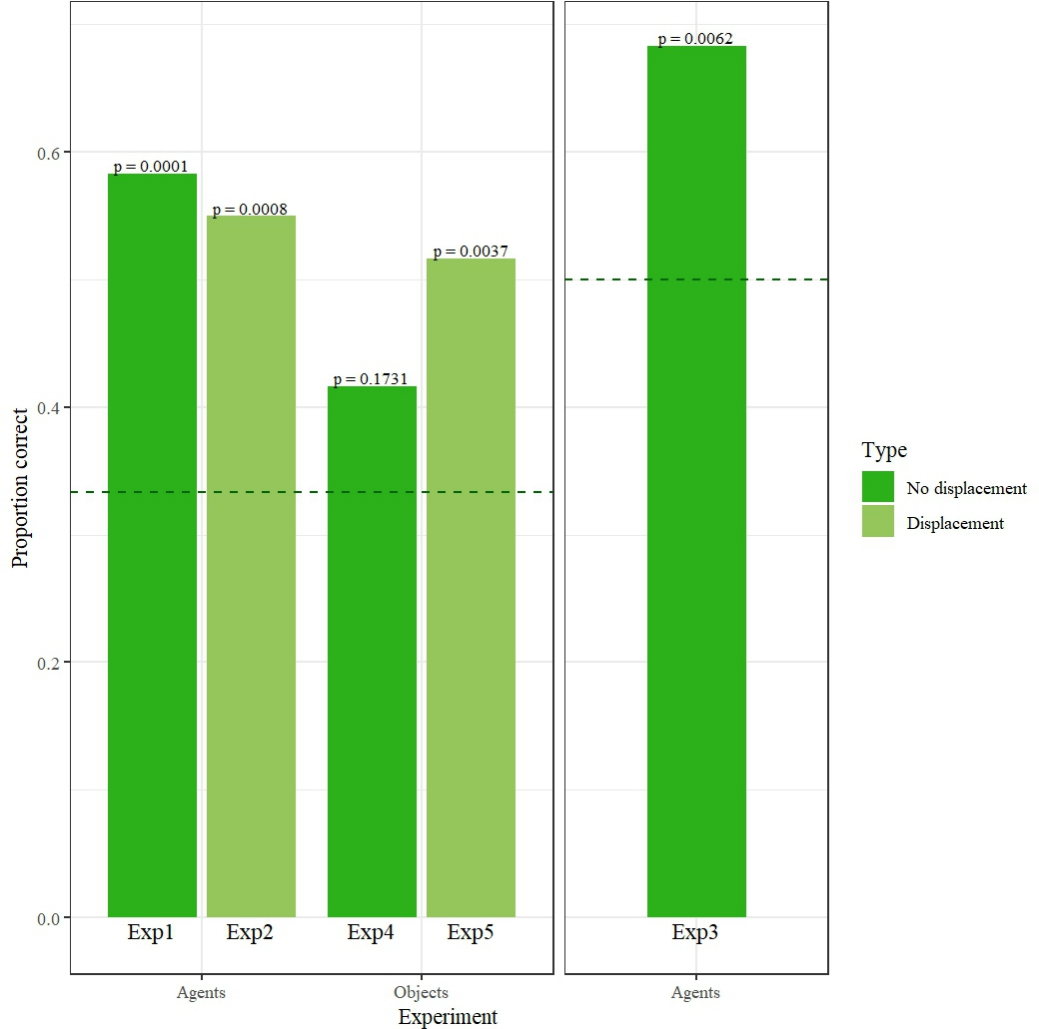
Kanzi’s proportion of correct responses in Experiments 1−5. Separate panels indicate experiments with different chance levels, and dotted lines indicate chance performance (33% for Experiments 1, 2, 4 and 5; 50% for Experiment 3). Corresponding *p*-values for binomial tests are shown on top of each bar.

Kanzi pointed at the location containing the correct person in 35 of 60 trials, significantly higher than if he had chosen randomly (two-tailed binomial test, 95% CI = 0.44–0.709, *p* < 0.001, test proportion = 0.33), indicating that he was able to remember the location of hidden agents. Kanzi’s frequency of responses for all experiments is reported in electronic supplementary material, table S1, and his performance across all experiments is depicted in figure 2. His performance cannot be explained by learning, as there was no improvement across trials (see table 1 for results, tested with a mixed effects logistic regression model with trial as a fixed effect, and further details in electronic supplementary material, table S2).

In principle, it may be possible to achieve reasonable performance by relying on one of several simpler strategies. Critically, however, each of these strategies makes clear predictions about choice or error patterns. We therefore directly tested each alternative account in follow-up analyses and found no support for any of them (see table 1 and electronic supplementary material, tables S2–S8). To test for a strategy where Kanzi was only tracking one person, we performed one separate binomial test for each experimenter, and found that he consistently tracked both of them. To test for a strategy where he would be tracking only one person (a different one each time) and then choosing randomly between the remaining two barriers, we inferred that this would show a pattern where most of his mistakes would be on the empty barrier: he would either happen to have tracked the person who was prompted, in which case he would be correct; or if the other person was prompted, he would be correct in half of the trials when he chooses randomly between the other two barriers, and only be wrong when the random choice leads him to the empty barrier. Thus we performed one-tailed binomial tests only on trials that were wrong, and found that the majority of errors were not on the empty barrier. To test for a strategy where Kanzi would remember which barrier was empty, ignore it and choose randomly between the other two, we performed binomial tests only on trials when he did not point at the empty barrier. Should he follow this strategy, this analysis would show performance at (50%) chance, which was not the case. Finally, if Kanzi was using a strategy of remembering which barrier was empty, ignoring it and tracking a single person; we propose that performance would be worse when the empty barrier was in the middle, as this makes objects harder to ignore [38], but we found no evidence for this either, using a logistic regression with ‘barrier being in the middle’ as a predictor. In conclusion, none of these alternative explanations are supported by Kanzi’s performance.

Taken together, our findings suggest that Kanzi was able to track the locations of multiple hidden agents. One strategy that cannot be discarded yet is that Kanzi was remembering the locations where he last saw each person, instead of actually mentally representing the agents as hidden behind those barriers. To dissociate the place where Kanzi last viewed each agent from the place where he should mentally represent them to be, in Experiment 2 we presented Kanzi with a hidden transposition. After the agents hid behind the barriers, the barriers were moved to the side, such that one barrier came to occupy the location of another (figure 1C).

#### (iii) Experiment 2: agent displacement

#### Methods

The procedure was identical to Experiment 1, except that once E2 and E3 were behind their corresponding opaque barriers, E1 moved all three barriers with E2 and E3 moving behind their barriers accordingly. As shown in figure 1C, during the initial hiding, the three barriers were relatively close together towards one side of the set-up, providing space for all three of them to be moved either toward the left or right (direction counterbalanced across sessions, to reduce unpredictability within sessions). In left movement trials, the left-most barrier was moved first (and furthest), followed by the central barrier, and finally the right-most barrier (with the opposite order in right movement trials). The second-moving barrier always ended up in the original baiting location of the first-moving barrier.

#### Results and discussion

Kanzi pointed at the location containing the correct person in 33 of 60 trials, significantly higher than if he had chosen randomly (two-tailed binomial test, 95% CI = 0.416–0.679, *p* < 0.001, test proportion = 0.33). We again found no evidence for learning or any of the alternative strategies (table 1, electronic supplementary material, table S3). These findings indicate that Kanzi was not simply tracking the last place where he saw the hidden agents. Instead, even through a transposition, he was mentally representing multiple agents at one time, as individuals with particular identities hidden behind particular barriers. To test if the representations that support his ability also incorporate auditory information, in Experiment 3 we tested Kanzi in an auditory recognition task where the only information he had about the location of the two people was their voices.

#### (iv) Experiment 3: auditory cues

#### Methods

This task followed the general procedure but involved only two barriers. A large opaque curtain was positioned in front of the two barriers, such that the space between the barriers could be entirely blocked when the curtains were drawn (see figure 1D). E2 and E3 simultaneously entered at the centre of the closed curtains, always entering from the same side regardless of which barrier they would ultimately occupy, and, once hidden, moved behind respective barriers. This procedure deprived Kanzi of any visual information about their ultimate whereabouts. Once the agents were in position, E1 opened the curtains to reveal the barriers (with E2 and E3 hiding out of sight behind them). Beginning always with the person on the left, E2 and E3 took turns saying ‘Hi, Kanzi.’ To assist Kanzi with localization of sound, as each agent spoke, E1 pointed at the barrier containing that agent. Because the vocalization always started from the left side, E1 always pointed at the left first, and the order of pointing was therefore not informative of the correct location of the person depicted in the photo prompt. Kanzi was then shown the photo prompt, and again heard E2 and E3 say ‘Hi, Kanzi,’ starting with the left. As in Experiments 1 and 2, E1 put down the photo prompt and said ‘Where is [E2/E3 name]?’, Kanzi then indicated his choice. If bonobos’ representations of individual agents contain auditory information, then Kanzi should be able to identify the location of the prompted individual based on her vocalizations alone. Because the prompt is still visual (printed photo) but the identity and location information is auditory (voice behind barrier), only through deploying a representation that integrates information from multiple sensory modalities can Kanzi answer the task correctly [40].

#### Results and discussion

Kanzi pointed at the location containing the correct person in 41 of 60 trials, significantly higher than if he had chosen randomly (two-tailed binomial test, 95% CI = 0.55–0.797, *p* = 0.006, test proportion = 0.5). Thus, Kanzi was able to connect a visual image of a familiar agent with her voice, and to identify her location based on this auditory cue alone. His performance cannot be explained by learning, as there was no improvement across trials (electronic supplementary material, table S4). Interestingly, Kanzi’s performance was better when prompted to find E2 than E3: he was correct in 25 of 30 E2 trials (two-tailed binomial test, 95% CI = 0.653–0.946, *p* < 0.001, test proportion = 0.5) but only 16 of 30 E3 trials (two-tailed binomial test, 95% CI = 0.343–0.716, *p* = 0.856, test proportion = 0.5). He knew both agents for roughly the same duration of time and had also successfully used the exact same photo prompts in Experiments 1 and 2, suggesting that he had a better capacity to identify the voice of E2 than E3. Kanzi’s failure to identify E3’s location above chance indicates that he was not simply excluding E2’s location in E3 trials but rather choosing randomly. This finding reinforces the interpretation that inference-by-exclusion did not drive Kanzi’s choices across experiments. Critically, however, he clearly showed a capacity to use auditory information to identify the location and identity of at least one agent. Given that Kanzi was able to track agents’ locations on the basis of either visual (Experiments 1−2) or auditory cues (Experiment 3), matched to an identical photo and verbal prompt, it is likely that he deployed cross-modal representations to solve the task. Kanzi’s success in Experiments 1−3, coupled with apes’ difficulty in past studies of multiple object tracking [26,27], raises a fundamental question about ape cognition: is there a distinct mechanism for agent representation, or was Kanzi’s performance improved by another aspect of the task (e.g. tracking entities with *different* identities, rather than pairs of identical entities). To discriminate these possibilities, in Experiments 4 and 5, we asked Kanzi to track the locations of two objects with different identities, in procedures that were analogous to Experiments 1 and 2.

#### (v) Experiment 4: hidden objects and Experiment 5: object displacement

#### Methods

The procedures of Experiment 4 and 5 were identical to Experiments 1 and 2, respectively, except for the following. We used much smaller barriers and two familiar objects (key and spoon) in place of agents (see figure 1E–F), and hiding of the objects behind the barriers was done by E1.

#### Results and discussion

In Experiment 4, Kanzi did not reach the threshold for significance, pointing at the location containing the correct object in 25 of 60 trials (two-tailed binomial test, 95% CI = 0.291–0.551, *p* = 0.173, test proportion = 0.33). It appears that Kanzi was only tracking the key, as he correctly pointed at the key in 17 of 30 trials (two-tailed binomial test, 95% CI = 0.374–0.745, *p* = 0.011, test proportion = 0.33), and at the spoon in eight of 30 trials (two-tailed binomial test, 95% CI = 0.123–0.459, *p* = 0.562, test proportion = 0.33). Notably, Kanzi began Experiment 4 after only two training sessions, in contrast to the 18 training sessions that preceded Experiment 1, and thus may have required more time to master the task. In line with this interpretation, Kanzi was successful at the more challenging Experiment 5 (which involved the hidden transposition). In Experiment 5, Kanzi pointed at the location containing the correct object in 31 of 60 trials, significantly higher than if he had chosen randomly (two-tailed binomial test, 95% CI = 0.384–0.648, *p* = 0.004, test proportion = 0.33). We found no evidence for learning (electronic supplementary material, Table S5) or the majority of alternative strategies discussed in table 1. However, in this particular experiment, we could not exclude the possibility that Kanzi was ignoring the empty barrier and choosing randomly between the two barriers containing objects. That being said, such a strategy is inconsistent with our finding from Experiment 4 that Kanzi was reliably tracking the location and identity of one of the objects (the key). Thus, it may be most parsimonious to conclude from Experiment 5 that Kanzi was mentally representing the locations and identities of both hidden objects, even after a hidden transposition. Future work should confirm this finding but if it is indeed the case, it would suggest that Kanzi can successfully track the whereabouts of multiple entities regardless of whether they were agents or objects. Accordingly, it would not be necessary to posit distinct mechanisms for multiple agent- and object-tracking. These capacities may recruit common representational machinery.

## 3. Discussion

Across five experiments, we have shown that Kanzi can simultaneously track the location and identity of at least two hidden agents (Experiments 1−2). This capacity seems to deploy multimodal representations of each agent that integrate visual and auditory signatures of identity, considering that Kanzi localized agents using either visual or auditory information matched to a (visual) photo prompt (Experiments 1−3). His relatively comparable abilities for multiple object-tracking (Experiment 5) are consistent with (though not diagnostic of) a common mechanism for mentally representing agents and objects.

The designs of Experiments 2 and 5 excluded the possibility that Kanzi was just remembering the location where he last saw an agent or object, revealing instead that he was actually representing them when they were out of sight, even through a hidden transposition. We found no evidence for learning, and our use of three barriers, rather than two, precluded reliance on an inference-by-exclusion strategy. Critically, we also tested a large number of alternative explanations in table 1, none of which predicted Kanzi’s performance in the agent-tracking tasks. His successful overall performance evidences a general capacity to mentally represent the locations and identities of multiple agents, and likely objects, at one time. This capacity is likely foundational for representing more complex phenomena, like social interactions, that occur out-of-view.

Past work has shown that great apes have the ability to remember where an object is hidden in an array of opaque cups, and to track the hidden motion of the object [25–30]. On the other hand, although chimpanzees have notable memory capacities [41], chimpanzees generally had more difficulty remembering the location of two hidden items (whether static or displaced [26,27]). Kanzi’s success in our tasks could derive from one of several key design differences. First, unlike past tasks, we required Kanzi to track entities with different identities, possibly making it easier to keep the two representations straight. Second, we required Kanzi to point at just one object, rather than both objects sequentially, likely reducing distractions and the duration of working memory demands. Finally, we used a smaller number of hiding places (three, rather than five or seven) that are more likely to fall within primates’ apparent object file capacity limits [42]. That said, a fruitful future endeavour would be to identify primates’ capacity limits for tracking agents and objects, a further test of the commonality of the underlying mechanisms. Future work could also individually manipulate each of the variables outlined here, to determine their influence on performance.

Cognitive studies with one or few participants are significant sources of information because, when conducted under a rigorous paradigm, they provide evidence of the cognitive potential of a species and show what a non-human mind can achieve, such as in other published work where Kanzi himself has been the sole participant [34,43]. Nonetheless, one important question concerns the generalizability of our findings to other bonobos. Kanzi provided a rare opportunity to study mental representation capacities because his extensive experience communicating via pointing and lexigrams and solving match-to-sample tasks allowed him to overcome the many ancillary demands of our task—and to directly communicate the content of his mental representations through his pointing behaviour. Strikingly, he had to use a photo prompt as a symbol for a live agent (or object), match the prompt to the barrier behind which he believed the entity to be, and point to communicate this choice—all to unveil his capacity to track agents or objects. The mere achievement of figuring out the task structure and understanding that the photo was a symbol referring to a real person in the room that needed to be pointed at is a significant cognitive challenge in itself [44,45].

That Kanzi was able to master the demands of our task meant that we could address our core question in a way that was much more direct and straightforward than would be possible with most other non-human apes. Critically, however, while Kanzi’s pointing and matching proficiency have likely been scaffolded by human interaction and language training, we do not believe that his mental representation capacities themselves are a product of his unique experience. Tracking the whereabouts of one’s affiliates should confer substantial adaptive value and, importantly, other studies provide evidence for the roots of this capacity (tracking single individuals) even in the wild [23]. In captive settings, it is plausible that apes’ motivation to track humans is dependent on the specific social dynamics of each location, though caregivers tend to be highly relevant social partners for apes. Kanzi was a highly motivated participant and, at Ape Initiative specifically, humans build long-lasting social bonds with the bonobos, and provide food and enrichment, making them worthy of tracking.

Although our task’s symbolic matching demands may be challenging for many non-human animals, the base task—using a familiar agent as a target in an object-choice context—may provide a highly motivating paradigm for future research into object and agent representation across diverse species. Indeed, many animals are able to point to or directly approach a barrier containing a hidden agent, and should be highly motivated to do so, whether to reunite with the agent (e.g. a caretaker or conspecific) or to receive a food reward from them.

Our work builds on seminal field experiments but makes new ground by demonstrating multi-agent tracking, likely reliance on cross-modal representations, and possible recruitment of a common system for representing agents and objects. Interestingly, field studies have found evidence of single agent-tracking in some species of Afro-Eurasian monkeys [23], but not others [24], raising questions about whether these mechanisms are a primate universal undetected in some paradigms, represent a deep homology that has been lost in some lineages, or have evolved multiple times through convergent evolution. The cost of losing groupmates in dense ecologies, and the benefits of being able to track their dynamics and social interactions, make this capacity highly adaptive and make either evolutionary story plausible. The adaptive value for bonobos in particular is underscored by observations that, despite splitting into distal parties during foraging [46], when encountering a leopard threat, their first response is to come together [47]. Future research should investigate whether primates represent not just small numbers of individuals in nearby locations but also map larger portions of the community across their territory, and what role the use of conspecific versus human agents plays in this process. Given that many social animals could benefit from multi-agent tracking capacities, future research should also investigate how widely shared these capacities are across species, and whether interspecific variation in traits like social complexity predict variation in representational limits or modality-independence.

Showing that a bonobo can solve tracking tasks by mentally representing hidden agents, contributes to our understanding of the cognitive adaptations that allow bonobos to skillfully navigate their social world. Past work shows that they can recognize members of their own group [13], develop individualized social relationships [48,49], and even remember familiar conspecifics for decades [12,14]. They also pay close attention to third-party interactions, and use this knowledge to inform their social decisions [50]. Much research is also consistent with a capacity to take others’ perspectives [51–53]. Our work adds to this picture by showing that bonobos’ representations of familiar individuals are likely to be multimodal in nature, that they can be mapped to physical space, and that multiple individuals can be represented at once. The agent and object representations documented here likely provide the foundations for building more complex representations of social interactions, relations, structures, and perhaps mental states. While our statistical analyses indicate that Kanzi is not infallible in his agent tracking ability, they nonetheless demonstrate this capacity. We conjecture that bonobos in the wild are likely even more motivated to track conspecifics in their day-to-day life, given potential fitness costs (e.g. if a separated individual or her offspring were predated) and potential time and energetic costs of finding lost groupmates in a spatially complex and large-scale territory. That being said, varied environments in the wild, as well as the challenge of tracking larger numbers of individuals, likely impose additional cognitive demands. Thus, future work should also investigate whether animals experience a representational limit in the number of agents that they can concurrently track, whether environmental complexity influences animals’ capacity to concurrently track multiple conspecifics, and whether animals deploy heuristics (e.g. relying on salient cues or prioritizing tracking of the most socially important individuals, like older females [54] or bond partners [14]) to optimize agent tracking.

This work uncovers the rich representations of the social world that are shared by humans and other apes. We have shown that a bonobo can mentally represent the identity and location of multiple (at least two) agents or objects on a short timescale. These representations likely undergird their navigation of both their social and physical worlds. That humans and our closest ape relatives share this representational machinery suggests that it was present at least 6−9 million years ago in our common evolutionary ancestor.

## Data Availability

All data and code are publicly available from OSF [55]. Supplementary material is available online [56].
